# An Examination of Factors Contributing to the Acceptance of Online Health Misinformation

**DOI:** 10.3389/fpsyg.2021.630268

**Published:** 2021-03-01

**Authors:** Wenjing Pan, Diyi Liu, Jie Fang

**Affiliations:** Journalism Department, School of Journalism and Communication, Renmin University of China, Beijing, China

**Keywords:** health misinformation, social media, misinformation acceptance, health-related anxiety, preexisting misinformation beliefs, repeated exposure

## Abstract

This study examined factors including health-related anxiety, preexisting misinformation beliefs, and repeated exposure contributing to individuals’ acceptance of health misinformation. Through a large-scale online survey, this study found that health-related anxiety was positively associated with health misinformation acceptance. Preexisting misinformation beliefs, as well as repeated exposure to health misinformation, were both positively associated with health misinformation acceptance. The results also showed that demographic variables were significantly associated with health misinformation acceptance. In general, females accepted more health misinformation compared to males. Participants’ age was negatively associated with health misinformation acceptance. Participants’ education level and income were both negatively associated with their acceptance of health misinformation.

## Introduction

Due to the development of technology and the popularity of social media, people are increasingly relying on the Internet as their primary source of information (Lazer et al., 2018). A similar trend has been observed worldwide as well as in China. Tencent’s WeChat application boasted a total number of monthly active users over 1.2 billion, and Sina Weibo reported daily 224 million active users as of the third quarter of 2020 (Statista, 2020). Social media has also become a prominent venue for people to exchange health-related information, which contributes to public health care, especially in China where many people possess insufficient health resources (Zhang et al., 2020). A Chinese national representative survey found that 98.35% of participants reported using WeChat to acquire health information (Zhang et al., 2017). Another study found that people’s personal beliefs were influenced by online information (Stevenson et al., 2007). At the same time, there are also critical concerns about the prevalence of misinformation on social media due to its potentially detrimental effects on individuals as well as on society (Broniatowski et al., 2018; Chou et al., 2018). For example, one study found that false news reached more people and spread faster than accurate news (Vosoughi et al., 2018). In an online knowledge contest hosted by the Food Safety Office of the State Council of China in 2019, 12 million participants answered 1,000 quiz questions with an accuracy rate of merely 50%. The spread of health misinformation has also been attributed as the most important reason for the low and inaccurate health and food safety knowledge (Xinhua News, 2019).

Misinformation has been widely discussed in the political realm, due to the significant impact of political news on socialization and democratization. For instance, by examining digital trace data across various electronic platforms, one study found that the audience of political fake news comprises a small, disloyal group of heavy Internet users (Nelson and Taneja, 2018). In recent years, misleading information was also observed in other topics such as vaccination and nutrition (Lazer et al., 2018), which pose great threats to public health.

Health misinformation refers to false or inaccurate health-related claims with a lack of scientific evidence (Chou et al., 2018). It inhibits individuals from engaging in health behaviors and diminishes institutional efforts to promote public health (Oh and Lee, 2019). Given its detrimental impact on the health of individuals, false statements have drawn attention from academia, industry, and governments (Chen et al., 2018). For example, several studies have explored the dissemination of specific health-related misinformation on social media, including cancer-related misinformation, Zika virus rumors, food safety-related rumors, and about influenza vaccines, etc. (e.g., Bode and Vraga, 2018; Chen et al., 2018; Oh and Lee, 2019). By analyzing tweets (posted on the social media platform Twitter) during the 2014 Ebola pandemic, researchers found that 10% of messages contained false, or at least partially false information (Sell et al., 2020). Another experiment focusing on the impact of conspiracy cues in Zika-related information found that conspiracy theories significantly influenced individuals’ beliefs, but exerted less tangible influence on people’s intentions to get vaccinated (Lyons et al., 2019).

Most existing literature is focused on the content characteristics of health misperceptions (Moran et al., 2016), diffusion patterns, and factors contributing to the fast spread of health misinformation (Chen et al., 2018; Jamison et al., 2020), as well as the impact of unsubstantiated statements (Broniatowski et al., 2018; Swire-Thompson and Lazer, 2020). Misinformation acceptance has been widely discussed in political psychology, and some scholars suggest that the psychological drivers that lead people to believe it needs to be addressed (Chou et al., 2020). Even though social media is an increasingly prominent way in which Chinese people acquire information about their health, a recent systematic review indicated that few studies examine the prevalence of misinformation in the Chinese context (Wang et al., 2019).

The convenience of social media sites makes it easy for ill-intentioned actors to purposefully disseminate misinformation, which can exert significant effects on individuals’ affective, cognitive, and behavioral responses regarding health-related issues. Thus, understanding the psychological mechanisms of people evaluating, processing, and trusting anti-science claims is crucial to constraining and debunking health misinformation. However, less is known about the role of belief systems, emotions, and cognition when it comes to people’s receptivity to specific health messages on social media.

This study details a large-scale online survey conducted through a mobile news application in China. The objectives of this study were to (a) examine how factors such as anxiety about health-related issues, preexisting beliefs, and repeated exposure to health misinformation may contribute to the acceptance of the unverified information on social media, and (b), to explore demographic differences (e.g., sex, age, education level, and social-economic status) in health misinformation acceptance.

## Factors Contributing to Health Misinformation Acceptance

Misinformation refers to “cases in which people’s beliefs about factual matters are not supported by clear evidence and expert opinion” (Nyhan and Reifler, 2010). Misinformation can be spread intentionally or unintentionally. Rumor is defined as “a proposition for belief in general circulation within a community without proof or evidence of its authenticity” (Walker and Blaine, 1991). In the current study, we do not intend to make a clear distinction between misinformation or rumor because the two terms both focus on the unique characteristics of unverified information.

Misinformation is not new to the Internet or social media. Nonetheless, Internet technology and the popularity of social media have facilitated the sharing of information as well as the prevalence of misinformation. The fast spread of misinformation on social media not only causes consequences such as invoking anxiety and fear among people but also leads to serious consequences such as forming irrational beliefs or undesirable behaviors (Fernández-Luque and Bau, 2015). This is especially the case for health-related misinformation. For example, even after corrections, 20–25% of the public continue to believe rumors regarding the association between childhood vaccines and autism (Lewandowsky et al., 2012).

In terms of factors contributing to the adoption of misinformation, some studies have focused on demographic differences such as sex, age, education level, or income, with the assumption that some people are naturally more inclined to believe misinformation (e.g., Greenspan, 2008). Other studies have focused on the content and the context of misinformation, suggesting that anyone can be susceptible to misbeliefs (Greenhill and Oppenheim, 2017). Research also showed that individuals rely upon sources, narrative, and contexts to evaluate the believability of social media messages (Karlova and Fisher, 2013).

Specific to health misinformation, some studies have explored the associations between psychological characteristics and people’s acceptance and trust in health misbeliefs. For instance, it was noted that individuals’ epistemic beliefs influence the extent to which they accept and share online misinformation (Chua and Banerjee, 2017). Online falsehoods can also prime individuals’ psychological proximity to health threats, and, in turn, the consequent higher perceived threats were found to increase the likelihood that people share misinformation (Williams Kirkpatrick, 2020). It was also discovered that exposure to collective opinion could reduce the propensity for individuals to believe and share health-related misinformation (Li and Sakamoto, 2015). Notably, in simulated pandemic settings, researchers found that the congruence between people’s original affective states and the emotions triggered by the contents led to higher acceptance of inaccurate claims (Na et al., 2018). Through interviews with people in the Hubei province of China during the COVID-19 outbreak, it was found that participants seldom seek confirmation of information. Even among those who strived to conduct verification, they relied more on heuristic cues rather than systematic processing strategies (Zou and Tang, 2020).

In the current study, we examine some key variables such as health-related anxiety, preexisting beliefs, and repeated exposure to misbeliefs in contributing to misinformation acceptance in the health arena. We then examine the associations between different socio-demographic variables and people’s health misinformation acceptance.

### Health-Related Anxiety

In the context of health misinformation perception, health-related anxiety can be defined as “how anxious the individual is about issues directly related to the rumor” (Greenhill and Oppenheim, 2017). Individuals who have higher levels of anxiety are more motivated to seek information to either legitimize their anxiety or to help them to ease the worry (Fergus, 2013). Previous studies have found a positive association between health anxiety and a need for health information (for a review, see McMullan et al., 2019). Considering that health information shared online often comes from unknown sources with low quality, people would spend more time to (dis)confirm the validity of the information (Starcevic and Berle, 2013). Under such circumstances, anxious individuals are less likely to use central processing cues such as examining the logic or the plausibility of the unverified information. Negative affective states such as fear and anxiety can even reinforce people’s believability of false information. Individuals who have higher levels of health anxiety were more affected by negative information and therefore more likely to share health misinformation (Pezzo and Beckstead, 2006; Cisler and Koster, 2010). For instance, one study noted that anxious people were more likely to disseminate misinformation as a way to vent negative emotions (Pezzo and Beckstead, 2006). Thus, the first hypothesis can be predicted:

**H1:** Individual’s health-related anxiety is positively associated with their health misinformation acceptance.

### Preexisting Misinformation Beliefs

Preexisting beliefs refer to people’s pre-existing opinions and attitudes they already have, before they process any information (Ecker et al., 2014). Persuasion literature indicates that individuals are more likely to accept messages which are congruent with their preexisting attitudes or opinions (McGuire, 1972). When individuals are presented with a piece of new information, they check it against their preexisting knowledge to assess its compatibility. Social judgment theory posits that persuasive messages are usually judged against an individuals’ existing position (anchor attitude). If the new message falls into the latitude of acceptance, then it will be perceived as closer than it is to the anchor attitude (Brehmer, 1976). Messages that are consistent with one’s beliefs will be evaluated and processed more fluently. Therefore, individuals are more likely to accept these messages as truthful. Messages which are not congruent or which contradict an individuals’ preexisting beliefs can cause cognitive dissonance and are more difficult to accept (Greenhill and Oppenheim, 2017).

In the context of health misinformation, individuals are more likely to believe a piece of new information if it is compatible and congruent with their preexisting beliefs. On the one hand, some people hold skeptical views when it comes to health-related information or misinformation. On the other hand, other people are more receptive and are more likely to accept what they were told. Therefore, we predict:

**H2:** Individuals’ preexisting beliefs will be positively associated with their health misinformation acceptance.

### Repeated Exposure

In the case of spreading misinformation, individuals may have different levels of exposure. Some people may have heard something many times and others may never have heard of it at all. Previous research in cognitive science has demonstrated that prior exposure increased the perceived accuracy of a statement (for a detailed review, see Dechêne et al., 2010). Repeated exposure to (mis)information may trigger several cognitive processes as well as bias in information processing. A common account is that repetition leads to the experience of processing fluency, which is known to shape illusory truth (Wang et al., 2016). For example, repeated exposure to the same information may increase an individuals’ perceived familiarity with the information, which makes it easier to process at both perceptual and conceptual levels (Whittlesea, 1993). The availability heuristic also states that people will use the most familiar and most recent information to serve as their base to form attitudes and infer the accuracy of certain statements. In particular, the effect of repeated exposure has also been detected in people’s trust in false and unverified information (Fazio et al., 2015). Misinformation can distort an individuals’ memory in that they may forget sources and over time, they morph the fictional sources into factual sources (Shen et al., 2019). Therefore, it is worth assuming that individuals will be more likely to believe health-related misinformation when it has been repeated.

**H3:** Individuals’ repeated exposure to health misinformation is positively associated with their health misinformation acceptance.

### Demographic Factors

The above-discussed factors may contribute to more or less acceptance of health misinformation. However, demographic characteristics may also affect how people respond to health misinformation. However, the existing literature remains inconclusive on how demographic factors such as age, sex, income, and education level may contribute to rumor acceptance. Therefore, we used a research question to examine the relationship between demographic characteristics and health misinformation acceptance.

RQ1: How might individuals’ sex, age, education level, and income associate with their health misinformation acceptance?

## Methods

### Participants and Procedure

A sample of 22,706 participants with a mean age of 31.13 (SD = 9.51) participated in the survey. Of these participants, 69.7% (*n* = 15,836) were female and 30.3% (*n* = 6,870) were male. Descriptive statistics on participants’ demographic information can be found in Table 1. The survey received ethical approval from the Internal Review Board from the School of Journalism and Communication, Renmin University of China. An online survey was conducted through Tencent News mobile application, as 99.1% of Internet users use mobile phones to access the Internet. Tencent is one of China’s largest Internet portals. In 2010, Tencent launched Tencent News application to provide users with the latest news and information. According to the statistics provided by Aurora Mobile, Baidu APP, Toutiao, and Tencent News APP are the biggest three news platforms with the largest numbers of daily active users to the hundred million volume level. Ranked as the second, Tencent News App reported a penetration rate of 22.4% as of the third quarter of 2020 (Sina News, 2020). The online questionnaire was randomly sent to users of Tencent News Mobile Application in mainland China. Consent was acquired before participants started answering the questionnaire. The survey questionnaire is included in Appendix A.

**TABLE 1:** Demographics of participants.

Demographic	Participants
Sex, *n* (%)	Female: 15,836 (69.7%)
	Male: 6,870 (30.3%)
Age	Mean = 31.13
	SD = 9.51
	Range: 24–70
Education level	Less than high school degree: 8,549 (37.7%)
	Associate degree in college: 6,732 (29.6%)
	Bachelor’s degree in college: 6,323 (27.8%)
	Master’s degree and above: 1,102 (4.9%)
Monthly disposable income	Less than 2,000 yuan: 3,550 (15.6%)
	2,001–5,000 yuan: 8,403 (37%)
	5,001–10,000 yuan: 6,748 (29.7%)
	More than 10,000 yuan: 4,005 (17.7%)

**FIGURE 1:**
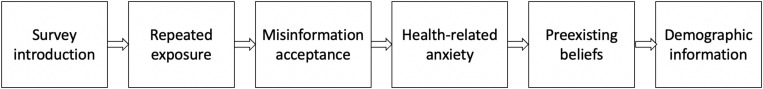
Survey design.

Participants were first presented with ten health-related items of misinformation. The criteria for misinformation selection in this study were based on the misinformation debunking messages released by the Jiaozhen fact-checking platform of Tencent News. The final misinformation items used in this study were selected based on popularity, frequency, and topics of misinformation. Participants were first asked to indicate whether they were aware of this misinformation item. They were then asked whether they believed it. After exposure to all ten pieces of health misinformation, they were asked about health-related anxiety, preexisting misinformation beliefs, and demographic information. The survey design can be found in Figure 1.

### Measures

#### Misinformation Acceptance

Misinformation acceptance was measured by asking participants to evaluate whether health-related (mis)information was true or not. Sample items include “Using microwaves to heat food may cause cancer” and “Eating catfish can lead to a terrifying disease called Halff’s disease, which is so difficult to treat that it has taken over the world.” Misinformation acceptance composite was calculated by aggregating the number of participants’ evaluations of rating the rumor’s as truthful out of the ten pieces of health misinformation (α = 0.85).

#### Health-Related Anxiety

Health-related anxiety measured how anxious participants were in evaluating their current health environment and future health conditions. To measure participants’ health-related anxiety, we adapted one question from a previous study (“How likely do you think you will encounter health-related issues in the future year,” Greenhill and Oppenheim, 2017) and added two more questions to form a scale (see Appendix A). The three items on a five-point Likert-type scale showed acceptable reliability (α = 0.72).

#### Preexisting Misinformation Beliefs

Participants’ preexisting misinformation beliefs were measured by two items on a five-point Likert-type scale including “If this is my first time reading a message regarding a health-related issue, I intend to believe it as true” and “If I come across a health-related message in WeChat groups or WeChat moments, I intend to believe it as true.” The two items showed acceptable reliability (α = 0.73).

#### Repeated Exposure

Before asking the participants to indicate their acceptance of the rumor, they were first asked to indicate whether they had heard or read the (mis)information previously. Repeated exposure was measured similarly to previous studies, examining food safety rumors (Feng and Ma, 2019) and political or economic rumors (Greenhill and Oppenheim, 2017). Overall repeated exposure was calculated by aggregating the score over the ten pieces of health misinformation. The ten items showed acceptable reliability (α = 0.74).

## Results

On average, participants only identified less than four items of misinformation. The Mean score for participants who fail to recognize the (mis)information was 6.55 with a standard deviation of 3.06. Hierarchical regression analysis was used to test the hypotheses and answer the research question (see Table 2). To conduct the stepwise regression analysis, demographic variables including participants’ sex, age, education level, and income were first entered in the linear regression model.

**TABLE 2:** Hierarchical regression analysis results in misinformation acceptance as the outcome.

Predictors	Model 1		Model 2		Model 3		Model 4	
	β	*t*	β	*t*	β	*t*	β	*t*
Sex	0.12	18.78*	0.9	13.99*	0.08	13.44*	0.06	10.29*
Age	–0.04	−6.66*	–0.04	−5.82*	–0.03	−5.52*	–0.06	−9.29*
Education	–0.12	−16.80*	–0.14	−21.28*	–0.13	−19.83*	–0.14	−22.00*
Income	–.05	−6.76*	–0.05	−8.24*	–0.05	−8.62*	–0.06	−9.58*
Health-related anxiety			0.31	50.10*	0.19	28.36*	0.16	25.01*
Preexisting misinformation beliefs					0.30	45.14*	0.29	44.84*
Repeated exposure							0.16	25.76*
*R*^2^	0.03		0.13		0.20		0.22	
Δ*R*^2^	0.03		0.10		0.07		0.02	
*F* change	193.53*		2,510.00*		2,037.95*		663.40*	

*indicates *p <* 0.001.

Firstly, the results showed that participants’ health-related anxiety (*M* = 3.30, SD = 0.80) was positively associated with misinformation acceptance, β = 0.31, *t*(22,700) = 50.10, *p* < 0.001. Participants’ health-related anxiety explained a significant portion of the variance in the misinformation acceptance, Δ*R*^2^ = 0.10, *F*(1, 22,700) = 2,510.00, *p* < 0.001, after controlling for demographic variables. Therefore, H1 was supported.

Secondly, participants’ preexisting misinformation beliefs (*M* = 2.74, SD = 0.90) were also positively associated with misinformation acceptance, β = 0.30, *t*(22,699) = 45.14, *p* < 0.001. Participants’ preexisting misinformation beliefs explained a significant portion of the variance in the misinformation acceptance, Δ*R*^2^ = 0.07, *F* (1, 22,699) = 2,037.95, *p* < 0.001, after controlling for demographic variables and the effect of health-related anxiety. Therefore, H2 was supported.

Thirdly, participants’ repeated exposure to misinformation (*M* = 4.94, SD = 2.53) was also positively associated with misinformation acceptance, β = 0.16, *t*(22,698) = 25.76, *p* < 0.001. Participants’ repeated exposure to misinformation explained a significant portion of the variance in the misinformation acceptance, Δ*R*^2^ = 0.02, *F* (1, 22,698) = 663.40, *p* < 0.001, after controlling for demographic variables, the effect of health-related anxiety, and the effect of preexisting misinformation beliefs. Therefore, H3 was supported.

Furthermore, since repeated exposure of misinformation was matched to misinformation acceptance, we conducted further item-by-item correlation analysis for each health misinformation. The results showed that previous exposure to health misinformation was positively correlated with misinformation acceptance. For the ten pieces of misinformation, the correlation between repeated exposure and acceptance ranged from 0.09 to 0.32. Detailed results can be found in Table 3.

**TABLE 3:** Item-by-item correlation between repeated exposure and misinformation acceptance.

Misinformation	Correlation coefficient	Statistical Significance
Using microwaves to heat food can produce carcinogens.	0.21	*p* < 0.01
Eating catfish gives people a horrible disease called Hough’s disease, which is hard to treat and has taken over the world.	0.22	*p* < 0.01
Some unscrupulous vendors make huge profits by injecting watermelons with red and sweet additives.	0.32	*p* < 0.01
Any meat that has been kept in the fridge for more than 3 months is not edible.	0.28	*p* < 0.01
Someone died of H7N9 infection from eating cherries.	0.11	*p* < 0.01
Acidosis can cause a variety of diseases, and alkaline food supplements can restore health.	0.09	*p* < 0.01
Fried garlic can cause cancer.	0.24	*p* < 0.01
Frequent drinking of ice water and beverages can lead to kidney failure.	0.32	*p* < 0.01
According to a recent report on fruit and vegetable residues released by the United States, strawberries have remained the dirtiest fruit for many years in a row.	0.21	*p* < 0.01
Eating crabs and tomato together is equivalent to eating arsenic.	0.18	*p* < 0.01

*N = 22,706.*

In order to answer the research question, demographic variables such as sex, age, education level, and income were also included in the linear regression model. The results showed that participants’ sex (sex was dummy coded as: 0 = male, 1 = male) was significantly associated with misinformation acceptance, β = 0.06, *t*(22,698) = 10.29, *p* < 0.001. Participants’ age was negatively associated with misinformation acceptance, β = −0.05, *t*(22,698) = −9.29, *p* < 0.001. Participants’ education level was also negatively associated with misinformation acceptance, β = −0.14, *t*(22,698) = −22.00, *p* < 0.001, as well as their income level, β = −0.06, *t*(22,698) = −9.58, *p* < 0.001.

## Discussion

As technology is making it easier to disseminate information on the Internet and social media, misinformation continues to influence people to a large degree in terms of forming their attitudes and opinions on socio-political issues worldwide. In particular, the proliferation of health misinformation on social media has raised critical concerns, especially in the current global pandemic when we also need to fight against an infodemic (Zarocostas, 2020). Considering the significant effects on individuals’ physical and psychological health, it is thus important to examine psychological responses to health-related misinformation on social media, which have largely been overlooked in comparison to political misinformation.

The present study reported the findings from a large-scale online survey conducted on a news mobile application in China. Based on previous literature on misinformation receptivity, the current study identified three emotional and cognitive factors in contributing to health misinformation acceptance, including anxiety about health-related issues, preexisting misinformation beliefs, and repeated exposure. Furthermore, this study also found that demographic factors such as an individuals’ sex, age, income, and education level were all associated with their acceptance of health misinformation.

Above all, the results showed that individuals’ anxiety about health-related issues was strongly associated with their health misinformation acceptance. This finding was in line with previous studies identifying health anxiety in increasing the odds that people accept and share unverified information (Oh and Lee, 2019). Individuals with higher health anxiety were sensitive to negative information and were more likely to spread unsubstantiated messages (Pezzo and Beckstead, 2006; Oh and Lee, 2019). Widely spread health-related misinformation on social media usually contains fear appeals and uncertainties, and thus they were loaded with intense emotion to instigate anxiety and panic. The negativity bias effect posits that negative information and related negative emotions can have greater effects on individuals’ psychological states compared to positive or neutral information (Rozin and Royzman, 2001), the effect of which was intensified among people who used to be more anxious about health-related issues. Resonating with the role of emotional congruence in rumor acceptance (Na et al., 2018), our results indicated that when exposed to anxiety-inducing messages, individuals tend to accept them because of their potential (negative) influences on their health, and they might spread these messages to ease their worry.

Secondly, the findings suggest that individuals’ preexisting misinformation beliefs were positively associated with their acceptance of misinformation. Confirmation bias stated that individuals were more likely to favor information that is in line with their prior beliefs. This not only applies to situations in which individuals already hold misinformation beliefs, but it could also be applied to ambiguous situations in that individuals tend to interpret ambiguous information as supporting their prior beliefs. Our findings reaffirmed the role of belief systems in leading people to accept misinformation in the context of health. As people devote limited cognitive efforts to assessing the truthfulness of information (Zou and Tang, 2020), they rely on their cognitive experience, together with their affective responses (i.e., health-related anxiety) to assess new information. For example, previous studies examining political rumors have found that people’s general tendency to believe conspiracy theories and worldviews is associated with the rejection of scientific findings (Lewandowsky et al., 2013). Similar results have been observed by studies investigating Zika virus-related rumors (Al-Qahtani et al., 2016) and anti-vaccine (mis)information (Jolley and Douglas, 2014).

Similarly, repeated exposure to health misinformation was also positively associated with misinformation acceptance. A previous study found that misinformation that was “sticky” enough to be circulated could be disseminated widely and proliferate (Heath and Heath, 2007). Even among participants who had a more solid knowledge base, repetition might still lead to insidious misperceptions (Fazio et al., 2015), suggesting that subjective knowledge may not always be effective in constraining fluency heuristics. Our empirical result showed that repeated exposure increased receptivity to health misinformation. Notably, the item-by-item analysis (Table 3) implied that the implausibility of each statement might alter the extent to which people believe it to be true. For instance, given consumers’ concerns about food safety, statements such as “some unscrupulous vendors make huge profits by injecting watermelons with red and sweet additives” seem to be reasonable, and thus more likely to be accepted.

In terms of demographic differences, the results showed that compared to men, women were more likely to accept health misinformation. Participants’ age, income, and education level were all negatively associated with their health misinformation acceptance. These results suggested that individuals with a higher socioeconomic status (including a higher education level and higher income) were less likely to accept health misinformation compared to those from lower socio-economic groups. Many studies have tested the knowledge gap hypothesis and found that social class was an important factor in explaining why information dissemination was more effective on people from a higher socio-economic group. One meta-analysis found that the knowledge gap was not only exemplified on social and political topics, but also on health-science topics and international issues (Hwang and Jeong, 2009). The health-science knowledge gap could affect whether people with different socio-economic status adopt preventive health behaviors. People with higher socioeconomic status tend to have higher levels of health literacy and are more likely to adopt recommended preventive health behaviors. Previous studies have found that engagement in preventive behavior was strongly influenced by other socio-demographic variables such as socioeconomic status, age, and sex (Rosenstock et al., 1994). For example, knowledge about food safety issues is positively associated with individuals’ evaluation of the susceptibility and risk perceptions related to food safety concerns (Mou and Lin, 2014).

Overall, the present study extended the theoretical models of misinformation acceptance into the health arena by exploring three potential predictors: health-related anxiety, preexisting misinformation beliefs, and repeated exposure to misleading statements. Together, we suggest that congruence (in terms of both affective states and beliefs), and repetition are two significant mechanisms that lead people to misperceptions. In this regard, anti-science statements may exert influence by increasing perceived familiarity, and, in turn, increase the ease with which a new piece of information is processed (i.e., processing fluency). Much more serious is that the impact of misperceptions may be strengthened among people who used to be anxious about health risks. As a result, some brief interventions, such as the myth vs. fact approach (Schwarz et al., 2007), may fail, or even backfire. Even explicit warnings or retracting pieces of misinformation in news articles (Ecker et al., 2010) can not significantly safeguard against the availability heuristic and diminish long-term misperceptions.

These findings offer valuable insights into misinformation debunking for health practitioners. Firstly, public health authorities should take measures to increase people’s institutional trust, which might alleviate their health-related anxiety, and, in turn, decrease the odds that people are instigated by inflammatory misbeliefs. Secondly, in a sense to tackle the effect of cognition bias and repeated exposure, active inoculating (or prebunking) may increase people’s ability to recognize and resist false information, as confirmed in the case of COVID-19 (van der Linden et al., 2020). Repeated warnings may be efficient in weakening the continued influence of misinformation resulting from individuals’ psychological characteristics (Ecker et al., 2011). Priority should also be given to social media messages that seem more plausible, or ambiguous, which were found to be more acceptable after prior exposure. More fundamentally, this study indicates the significance and urgency of improving individuals’ health literacy, which enables people to critically examine health (mis)information on social media. The current study found that repeated exposure and preexisting misinformation beliefs were linked with people’s receptivity and the believability of false statements, yet both are uncontrollable in practice. To the extent that it is difficult to control how people expose themselves to unsubstantiated health claims, improved health literacy can nevertheless help individuals to better examine the plausibility of health (mis)information on social media and reduce uncertainty.

## Limitation and Future Direction

The current study also has several limitations. First, the health-related misinformation used in the study was by no means representative of all health misinformation on social media. It is still possible that the observed associations among the key variables only pertain to the specific health misinformation adopted in the current study. Although the topics we have included in this study were carefully selected by a fact-checking platform by popularity, frequency, and generalizability, future studies could incorporate various health-related topics with varying levels of severity and familiarity.

Second, the design of the study was correlational. Although significant associations were observed in the survey, causal relations can only be established by using longitudinal design or controlled experiments. Future studies could also use carefully designed experiments with pre-tested materials as stimuli to rule out the effects caused by other confounding factors. With the advancement in computational social science, future studies could adopt more sophisticated methods to collect individuals’ behavioral data together with individuals’ social network data to trace the actual spread misinformation while controlling for network effects.

Third, the measurements in this survey were mostly self-generated. Although the key variables we aimed to study were inspired by previous studies, considering the practical issues in survey distribution and logistics, we have included self-generated measurements to better fit the scope of the current study. Since the composites we calculated in the study all showed acceptable reliability, the self-generated measures do not undermine the practical contributions of these findings. Future studies could incorporate both subjective and objective measurements to measure individuals’ affective, cognitive, and behavioral responses to health misinformation. For example, in addition to anxiety or health misinformation acceptance, future studies could use measurements such as emotions, perceived trust, intentions to share, and actual sharing or disseminating behaviors.

## Conclusion

In the age of fake news and online misinformation, the negative effects of this dissemination cannot be overlooked. Health misinformation should receive more attention from both academia and industry due to its significant impacts on individuals’ lives. This article reported key results from a large-scale online survey aimed at dissecting what factors contribute to the acceptance of health misinformation. Factors including health-related anxiety, preexisting misinformation beliefs, and repeated exposure contributed to health misinformation acceptance. Furthermore, the results suggest obvious demographic differences in health misinformation acceptance, as female participants in this study were more likely to accept health misinformation as true compared to males, and individuals with a lower educational background and low-income were more receptive to health misinformation compared to those who received higher education and have higher incomes.

## Data Availability Statement

The raw data supporting the conclusions of this article will be made available by the authors, without undue reservation.

## Ethics Statement

The studies involving human participants were reviewed and approved by the Institutional Review Board School of Journalism and Communication Renmin University of China. The patients/participants provided their written informed consent to participate in this study.

## Author Contributions

WP and JF contributed to the conception and design of the study. JF organized the experiment. WP and DL performed the statistical analysis and wrote the first draft of the manuscript. All authors worked on revisions of the manuscript and performed the additional statistical analysis, wrote the sections of the manuscript, contributed to manuscript revision and, read the manuscript, and approved the submitted version.

## Conflict of Interest

The authors declare that the research was conducted in the absence of any commercial or financial relationships that could be construed as a potential conflict of interest.

## Appendix

Appendix A. Survey questionnaire.

**Table:** 1. Have you heard of the following information?

	Yes	No
Using microwaves to heat food can produce carcinogens.		
Eating catfish gives people a horrible disease called Hough’s disease, which is hard to treat and has taken over the world.		
Some unscrupulous vendors make huge profits by injecting watermelons with red and sweet additives.		
Any meat that has been kept in the fridge for more than 3 months is not edible.		
Someone died of H7N9 infection from eating cherries.		
Acidosis can cause a variety of diseases, and alkaline food supplements can restore health.		
Fried garlic can cause cancer.		
Frequent drinking of ice water and beverages can lead to kidney failure.		
According to a recent report on fruit and vegetable residues released by the United States, strawberries have remained the dirtiest fruit for many years in a row.		
Eating crabs and tomato together is equivalent to eating arsenic.		
		

2. Do you think the aforementioned information is true?			
	**Not true**	**Not sure**	**True**

Using microwaves to heat food can produce carcinogens.			
Eating catfish gives people a horrible disease called Hough’s disease, which is hard to treat and has taken over the world.			
Some unscrupulous vendors make huge profits by injecting watermelons with red and sweet additives.			
Any meat that has been kept in the fridge for more than 3 months is not edible.			
Someone died of H7N9 infection from eating cherries.			
Acidosis can cause a variety of diseases, and alkaline food supplements can restore health.			
Fried garlic can cause cancer.			
Frequent drinking of ice water and beverages can lead to kidney failure.			
According to a recent report on fruit and vegetable residues released by the United States, strawberries have remained the dirtiest fruit for many years in a row.			
Eating crabs and tomato together is equivalent to eating arsenic.			
			

3. How would you evaluate the health environment you live in?

• Very unsafe
• Somewhat unsafe
• Not sure
• Somewhat safe
• Very safe

4. Do you often worry about health-related issues?

• Strongly disagree
• Somewhat disagree
• Not sure
• Somewhat agree
• Strongly agree

5. How likely do you think you will encounter health-related issues in the future year?

• Very unlikely
• Somewhat unlikely
• Not sure
• Somewhat likely
• Very likely

6. Please use the scale to indicate to what extent do you agree or disagree with the following statements:

1 = completely disagree; 5 = completely agree

(1). If this is my first time reading a message regarding a health-related issue, I intend to believe it as true.

(2). If I come across a health-related message in WeChat groups or WeChat moments, I intend to believe it as true.

7. What is your sex?

• Male
• Female

8. What is your age? ____________

9. What is the highest level of school you have completed?

• Less than high school degree
• Associate degree in college
• Bachelor’s degree in college
• Master’s degree and above

10. What is your monthly disposable income (excluding tax, house mortgage, automobile mortgage, and day-to-day expense)?

• Less than 2,000 yuan
• 2,001–5,000 yuan
• 5,001–10,000 yuan
• More than 10,000 yuan
